# Multiple Facets of Value-Based Decision Making in Major Depressive Disorder

**DOI:** 10.1038/s41598-020-60230-z

**Published:** 2020-02-25

**Authors:** Dahlia Mukherjee, Sangil Lee, Rebecca Kazinka, Theodore D. Satterthwaite, Joseph W. Kable

**Affiliations:** 1 Penn State College of Medicine and Penn State Milton S. Hershey Medical Center, Department of Psychiatry and Behavioral Health, Hershey, USA; 2 University of Pennsylvania, Department of Psychology, Philadelphia, Pennsylvania USA; 3 University of Minnesota, Department of Psychology, Minneapolis, Minnesota USA; 4 Perelman School of Medicine, Department of Psychiatry, University of Pennsylvania, Philadelphia, USA

**Keywords:** Depression, Diagnosis

## Abstract

Depression is clinically characterized by obvious changes in decision making that cause distress and impairment. Though several studies suggest impairments in depressed individuals in single tasks, there has been no systematic investigation of decision making in depression across tasks. We compare participants diagnosed with Major Depressive Disorder (MDD) (n = 64) to healthy controls (n = 64) using a comprehensive battery of nine value-based decision-making tasks which yield ten distinct measures. MDD participants performed worse on punishment (*d* = −0.54) and reward learning tasks (*d* = 0.38), expressed more pessimistic predictions regarding winning money in the study (*d* = −0.47) and were less willing to wait in a persistence task (*d* = −0.39). Performance on learning, expectation, and persistence tasks each loaded on unique dimensions in a factor analysis and punishment learning and future expectations each accounted for unique variance in predicting depressed status. Decision-making performance alone could predict depressed status out-of-sample with 72% accuracy. The findings are limited to MDD patients ranging between moderate to severe depression and the effects of medication could not be accounted for due to the cross sectional nature of the study design. These results confirm hints from single task studies that depression has the strongest effects on reinforcement learning and expectations about the future. Our results highlight the decision processes that are impacted in major depression, and whose further study could lead to a more detailed computational understanding of distinct facets of this heterogeneous disorder.

## Introduction

Depression is the most debilitating disease in the world according to the World Health Organization (WHO)^1^. Decision processes are likely an important and understudied target for better understanding this heterogeneous disorder^2^. Many of the ways that depression reduces quality of life are through cognitive distortions and changes in decision making. A recent meta-analysis of decision making on the Iowa Gambling Task (IGT) demonstrated impaired decision-making performance across mental illnesses, including depression^3^. In addition, depression is associated with impaired functioning in ventromedial prefrontal cortex and ventral striatum^4,5^, two regions known to play critical roles in value-based decision making^6–8^. Using the conceptual and analytical tools of decision science to study depression could provide a more nuanced understanding of the processes and mechanisms affected by the disorder, shed light on the nature of heterogeneity within depression, and help bridge gaps between current research in clinical psychopathology and neuroscience^2,9–12^.

Though several studies have begun to investigate decision-making in depression, there are some noteworthy gaps in the literature. First, we do not have a full characterization of how value-based decision making differs in depressed individuals. Several studies have demonstrated differences in single tasks^2,13–16^, but no study has systematically investigated a wide range of different decision processes in the same depressed individuals. Therefore, it remains unclear which decision making domains are most impacted by depression. Here we address this gap by characterizing depressed and non-depressed individuals on ten different decision-making measures and comparing effect sizes across tasks. Second, whether differences reflect impacts on a single dimension of decision-making or multiple dimensions is uncertain. Answering this question requires looking across a range of decision tasks to identify the dimensionality of changes in decision making in depressed individuals. Here we address this issue using both factor-analytic and regression approaches to determine whether different decision tasks are associated with unique variance in depression. Finally, we do not know how well we can successfully categorize depressed from healthy individuals based on decision behavior. There is excitement about the emerging field of “computational psychiatry”^2,9,11^, including the prospects for using a combination of behavioral tasks and computational models to predict and classify disorders. How much of the depression “signal” can multiple measures of decision making detect? Here we address this question using machine learning techniques to perform out-of-sample predictions of depressed status based on decision-making performance.

Accordingly, we performed a comprehensive evaluation of decision making in depression using a battery of nine tasks (yielding ten measures) that spanned different domains of choice behavior. Though an exhaustive global assessment of value-based decision making would be impossible, we chose tasks to span the major of dimensions of decision making that have been related to neural function and putatively linked to psychopathology, including temporal decision-making, choices under risk and uncertainty, social decision making, and reward and punishment expectations and learning^12^. Within each domain, we selected tasks for which there was some evidence of performance differences in depression^13–15,17–25^. In the time domain, we assessed both prospective willingness to wait for delayed rewards using a delay discounting task^26–28^ as well as experiential willingness to wait with a persistence task^29^. In the uncertainty domain, we measured tolerance for both risk^30,31^ and ambiguity^16,32^. In the social domain, we assessed bargaining in the ultimatum game as both proposers and responders^14,18,33^. Finally, in the learning domain, we tested learning in response to both rewards and punishments^34,35^, as well as expectations about future rewards^36,37^.

To foreshadow our results, we find that reward and punishment learning, future expectations and persistence are the aspects of decision making most strongly affected in depressed individuals, that these tasks reflect independent dimensions of decision making, and that these tasks combined predict depressed status out of sample at above 70% accuracy.

## Methods

### Participants

The study was approved by the University of Pennsylvania Institutional Review Board (IRB # 815189) and all participants provided informed consent. All methods were performed in accordance with the approved IRB guidelines and regulations. Between October 2012 and January 2014, 128 participants (64 diagnosed with current Major Depressive Disorder (MDD) and 64 matched healthy controls) were recruited for the study. MDD participants were recruited from the Department of Psychiatry and Behavioral Health, the Counseling and Psychological Services and the Hospital of the University of Pennsylvania. Healthy control participants were recruited from the University of Pennsylvania and local community through flyers. Based on an initial phone screen for depression, participants were invited for a diagnostic interview. MDD participants were enrolled if they (1) met diagnostic criteria for current MDD; (2) had no history of substance abuse/dependence in the past 6 months; and (3) had no history of bipolar disorder and/or psychotic episodes. Potential comorbidities beyond bipolar, substance use or psychotic disorders were not assessed. A master’s level clinical psychologist determined diagnostic criteria based on the mood sections of the Structured Clinical Interview for *Diagnostic and Statistical Manual of Mental Disorders, Fourth Edition* (SCID/DSM-IV)^38^. Fifty-six percent (36 out of 64) of the MDD participants were also referred directly from another clinical study or clinical service based on an MDD diagnosis. Inclusion criteria for controls included absence of current or past psychiatric illness, as assessed by the SCID, and absence of any psychotropic medications. Within the MDD group, 10 were on medication only, 14 were receiving therapy only, 19 were receiving both medication and therapy, and 19 were in no treatment for depression.

### Measures

#### Self-report measures

Participants completed the Rosenberg Self-Esteem Scale^39^, the Cognitive Behavioral Avoidance Scale^40^, the Behavioral Inhibition and Behavioral Activation Scale^41^, the Snaith Hamilton Pleasure Scale^42^, the Beck Depression Inventory-II (27), the Beck Anxiety Inventory^43^ and the Depression, Anxiety and Stress Scale^44^.

#### Cognitive measures

Two subtests (matrix reasoning and similarities) of the Wechsler Abbreviated Scale of Intelligence - Second Edition^45^ were used as a brief, reliable measure of cognitive ability.

#### Value-based decision making tasks

Participants completed a battery of nine decision-making tasks assessing risk tolerance, ambiguity tolerance, delay discounting, persistence, reward and punishment learning, social bargaining in the ultimatum game, and future reward expectations. The persistence task yields two measures, for a total of ten decision-making measures across the task battery.

### Decision task details

#### Risk tolerance task

This task was used to measure a participant’s degree of risk tolerance. The task consisted of 51 choices. In each trial, participants were presented with the option of choosing either a smaller guaranteed amount of money or a 50% chance of winning a larger amount of money. For example, “would you choose a 50% chance of winning $35 or a sure amount of $5?” The expected value of the risky option (probability of winning times amount) could either be higher or lower than the safe amount. In this task as well as the ambiguity tolerance and delay discounting tasks, participants had two practice trials beforehand, and had a 10-second limit to indicate their choice on each trial. Performance was measured in percentage of safe options chosen. We did this for the present report to focus on the metrics that make the least assumptions, but we observed similar effects with more theoretically driven measures for risk aversion (e.g., power utility function exponent).

#### Ambiguity tolerance task

This task was used to measure a participant’s degree of ambiguity tolerance. The task consisted of 66 choices. In each trial, participants were asked to choose between a gamble where the chances of winning are known to be 50% (risky) versus a gamble where the chances of winning are not known exactly (ambiguous). Participants were informed that ambiguous probabilities were the same for each trial. The amount won for the risky gamble was always equal to or lesser than the ambiguous gamble. Performance was measured in percentage of risky gambles chosen.

#### Delay discounting task

This task was used to measure the extent to which a participant discounts delayed rewards. The task consisted of 51 choices. Each choice was between a smaller monetary reward available immediately and a larger monetary reward received after a delay. For example, “Would you prefer $10 now or $15 in 7 days?” Performance was calculated in percentage of now options chosen. We did this for the present report to focus on the metrics that make the least assumptions, but we observed similar effects with more theoretically driven measures for delay discounting (e.g., discount rates).

#### Persistence or willingness to wait (WTW) task

In the persistence task, decision makers faced the problem of optimizing persistence (in the form of waiting for a higher monetary reward) appropriately to their environment. The task was divided into two blocks. In the first block, unlimited persistence provided the highest return, whereas in the second block, continued persistence if the reward has not arrived by a certain point was suboptimal. Participants could choose to wait by leaving the mouse cursor in a box marked, “Wait for 30¢”. Alternatively, by shifting to a box marked “Take 2¢”, participants could receive 2¢ and proceed to a new trial. Each outcome (30¢ or 2¢) was followed by a 2-sec inter-trial interval (ITI). The cursor could remain in either box across multiple trials. The task was divided into two blocks, each lasting seven minutes (total duration 14 min), and the screen continuously displayed the time remaining and total earned. The optimal strategy for the first block was to always wait for the 30¢, while the optimal strategy for the second block was to wait for the 30¢ reward for 5 secs and then take the 2¢ if the large reward had not arrived.

Individual trials provide different amounts of information about a participants’ willingness to wait. Quit trials are the most informative, providing a direct estimate of the limit on a participant’s willingness to persist. When the reward is delivered, however, we observe only that the person was willing to wait *at least* the duration of the trial. We accommodate this situation using statistical methods from survival analysis. Analyses assessed how long a trial would “survive” without the participant quitting.

We constructed a Kaplan-Meier empirical survival curve from each participant’s responses. For each time *t*, the curve plots the probability of the participant waiting at least until *t*, provided that the reward was not delivered earlier. As participants could keep the cursor in the “Take 2¢” box across trials, this analysis only included trials where participants initially indicated a preference for the “Wait for 30¢” box (cursor remained there for >1 s). Analyses were restricted to the 0–16 sec interval common to the two conditions. The area under the survival curve (AUC) is a useful summary statistic, representing the average number of seconds an individual was willing to wait within the analyzed interval. Someone who never quit earlier than 16 sec would have an AUC of 16. One who was willing to wait up to 4 sec on half the trials and up to 12 sec on the other half would have an AUC of 8.

#### Reward learning task

In this task, participants chose between two distinct fractal stimuli, which were positioned randomly across trials on the left or right of the screen. On each trial, participants responded by pressing a button on a keyboard to choose a fractal. The fractals were probabilistically rewarded; with the “richer” fractal rewarded 70% of the time and the “poorer” fractal rewarded 30% of the time. Positive feedback was provided if a fractal is rewarded (picture of a coin); otherwise, neutral feedback was provided (a red dot; indicating no coin). Participants were not informed of the specific underlying reward structure of the task. However, they were informed that on any given trial, one fractal had a higher likelihood of delivering a reward and that this association reverses periodically throughout the task. All participants completed 4 trials as practice before proceeding to do a full run of 90 trials. Switches took place after 30 trials; hence, there were two switches in total. Each reward had a monetary value of 25¢. At the end of the task, the screen displayed the total number of quarters the participant won. In this task, we calculated the proportion of trials the participant chose the richer fractal image (i.e., had the higher probability of positive feedback).

#### Punishment learning task

This task is similar to the reward learning task, except the goal for the participant was to avoid choosing the fractal leading to punishment feedback (red cross overlaying a coin). Participants were informed that at any given point, one fractal image lead to more losses than the other and that this will switch periodically. The participants started the task with $22.50 and each time they chose the fractal image followed by punishment feedback, 25¢ was deducted from the total amount. Like the reward task, participants did a 4 trial practice, before proceeding to do a full run of 90 trials, with two reversals. On completion of the task, the screen displayed the total number of quarters the participant lost. Again, we calculated the proportion of trials the participant chose the richer fractal image (i.e., had the higher probability of no-punishment feedback).

#### Ultimatum game, proposer

Participants were instructed that the game involved two people; the participant was the proposer and an anonymous person was the responder. They were informed the anonymous responder was a real person whose responses had already been recorded. As the proposer, the participant had $10 and could propose to divide this money as s/he wished between himself/herself and the anonymous responder. The anonymous responder had the right to either accept or reject the proposal. Should the proposal be accepted, the money would be divided the way the proposer decided. However, if the proposal was rejected, neither the proposer nor the responder received any money. The amount of money the proposer decided for him/herself was recorded. Participants completed a practice quiz to ensure they understood the rules of the game.

#### Ultimatum game, responder

Participants also played the responder’s role in the ultimatum game. The participant decided whether s/he would accept or reject each possible proposal an anonymous proposer could make. Should the participant accept the proposer’s division, the money would be divided accordingly. They were informed that the proposer was a real person whose responses had already been recorded. The minimum amount of money the participant was willing to accept was recorded.

#### Prediction question

Each participant responded to this questionnaire at the end of the study, prior to knowing whether or not they received additional payment. The questionnaire states, “You have now completed all the required tasks. One item will be randomly picked from one of the tasks you have performed. You may or may not receive additional money based on your response. What do you think are the chances that you will win additional money (in addition to the $30 for participation) on a scale of 0–100%? If you think you have an above 0% chance of winning, then how much do you think you will win in the range of $0–$100?” We used the answer to the first question to assess the accuracy of participants’ future reward expectations.

### Procedure

Participants received $30 for the two-hour study and an additional payment based on their responses to one of eight of the decision tasks (excluding future expectations), to ensure that these tasks had real consequences for the participants. The task chosen for additional payment was randomly selected, and participants were informed of the payment procedures prior to performance of each task. The sequence of administration was: (1) the structured clinical interview, (2) eight of the decision-making tasks, with the order counterbalanced across participants according to a Latin-square design, (3) the similarities and matrix reasoning subtests of the WASI; (4) seven self-report questionnaires; and (5) the ninth decision-making task, which consisted of two questions assessing expectations about winning additional money from the previous eight decision-making tasks.

### Statistical analysis

Statistical analyses were performed using SPSS 21.0 and MATLAB 8.2. All tests were two-sided. We compared groups on demographic and clinical characteristics using independent *t*-tests, one-way ANOVA and chi-square tests. Effect sizes for performance differences between the two groups on each decision task were calculated and their significance evaluated with independent *t*-tests.

#### Factor analysis

To determine underlying latent factors captured by behavioral performance on decision-making tasks, we used principal axis factoring using promax rotations, which allows factors to be correlated. The common rule of thumb for power is including at least 10 subjects/cases per item/instrument being analyzed^46^. The present factor analysis included more than 10 subjects per task. We examined the scree plot to confirm the number of factors. The data were screened for univariate outliers. With a final sample size of 115 (using listwise deletion), the minimum amount of data for factor analysis was satisfied, with a ratio of over 11 cases per variable. The Kaiser-Meyer-Olkin measure of sampling adequacy above the recommended value of 0.5 and Bartlett’s test of sphericity was significant (χ^2^ (45) = 146.72, *p* < 0.001). The diagonals of the anti-image correlation matrix were all over 0.5.

#### Logistic regression

To determine the relative predictive power of different decision tasks, logistic regression was conducted with factor scores and with task scores for the different decision tasks. For the task score regression, we report a full regression model using all measures, as well as a reduced model constructed using the backward stepwise selection method. This method starts with all predictor variables in the model, deleting the predictor (if any) that improves the model the most, and repeating this process until no further deletions improve the model. We focus on predicting depressed status, rather than depression severity; given the nature of our design, there is no overlap in depression severity across groups, and thus depression severity and depressed status are highly colinear.

#### Out-of-sample prediction of depressed status

To examine how well depressed status could be predicted from decision-making tasks out-of-sample, we used a boosted decision trees algorithm to perform leave-one-subject-out cross validation (*i.e*., a model was fit to 127 participants and then used to predict the left-out participant’s clinical label). We selected boosted trees as it allows for non-linearities and interactions between variables and is known to handle missing data well via the surrogate split technique. We used AdaBoost as the fitting method with a parameter configuration of 100 tree learners, each with a maximum of 1 node split and a regularization learning rate of 0.1. To assess variable importance, we fit the entire dataset with the same parameter configurations and calculated the change in classification scores with and without a given variable.

#### Correlations between decision-making and self-report measures

We assessed Pearson’s correlations between each task and each of the self-report measures described above controlling for group (control vs MDD).

## Results

### Demographic characteristics

The groups were similar in their demographic characteristics (*p* values> 0.30, Table 1) and did not differ in cognitive ability (*p* = 0.35, *t* = 0.94). While matching on cognitive ability may mean that our MDD group is not representative of MDD as a whole, it permits us to test the relationship between MDD and decision-making without cognitive confounds that are known to impact decision-making^47,48^. As expected, the MDD group had higher BDI-II scores (*p* < 0.001, *t* = 19.33, *d* = 3.42). Participants in the MDD sample were mildly to severely depressed, with a mean score of 30 (SD = 10.5) on the BDI-II and scores ranging between 12 to 51.

**Table 1:** Demographic and Clinical Characteristics of MDD and Healthy Control Groups.

	Depressed		Control		Significance
	N	%	N	%	*p* value
Gender					0.3
Male	27	57.8	33	51.6	
Female	37	42.2	31	48.4	
Ethnicity					0.76
African American	34	53.1	33	51.6	
Caucasian	24	37.5	23	35.9	
Asian	4	6.2	7	10.9	
Other	2	3.1	1	1.6	
Education					0.31
No High School Diploma	4	6.3	1	1.6	
High School Training	26	41.3	23	35.9	
Associate’s Degree	10	15.9	12	18.8	
Bachelor’s Degree	12	19	16	25	
Master’s Degree	11	17.5	9	14.1	
Doctoral Degree	0	0	3	4.7	
Depression Treatment					<0.001**
No	19	29.7	60	95	
Medication Only	10	15.6	0	0	
Therapy Only	14	21.9	0	0	
Both	19	29.7	0	0	
Missing	2	3.1			
	M	SD	M	SD	*p* value
Age	40.45	13.48	38.53	11.73	0.4
WASI	101.32	14.63	103.7	14.1	0.35
BDI	30.03	10.46	2.9	4.32	<0.001**

*Note*. **p < 0.001.

### Value-based decision making

For four decision-making measures, there were medium effect size differences between the MDD group and healthy controls, with confidence intervals that did not cross zero (Table 2). The largest differences were in punishment reversal learning (*d* = −0.54, *t* = 3.10, *p* = 0.003), where depressed participants made fewer choices of the more advantageous (“rich”) option. Slightly smaller differences of the same direction were also observed in reward reversal learning (*d* = −0.38, *t* = 2.15, *p* = 0.03). The second largest differences were on the future expectations questionnaire, where depressed participants expressed a lower expectation of winning additional money (*d* = −0.47, *t* = 2.54, *p* = 0.01). Note that the objective odds of winning additional money were similar for the depressed and control groups and the depressed group earned on average an extra $34.56 (SD 27.27) while the control group an extra $30.77 (SD 21.51). The third largest differences were in the high persistence condition of the willingness-to-wait task, where depressed participants showed a reduced willingness to wait when the optimal strategy was to persist until the reward arrives (*d* = −0.39, *t* = 2.22, *p* = 0.03). The *p*-values reported above are uncorrected for multiple comparisons; the differences in punishment learning and future expectations remain statistically significant when controlling for multiple comparisons across ten tasks using the false discovery rate.

**Table 2:** Decision Task Performance In MDD and Healthy Control Groups.

Decision Task	Measure	Depressed			Control			p value	Cohen’s d	95%
		M	SD	N	M	SD	N			CI
Risk Tolerance	Safe Choices (%)	57.03	23.04	64	59.12	22.34	64	0.60	−0.09	−0.44 to 0.26
Ambiguity Tolerance	50–50 Choices (%)	60.30	26.58	64	67.34	25.71	64	0.13	0.27	−0.62 to 0.08
Reward Learning	Rich Choices (%)	59.10	9.50	64	62.98	10.91	64	0.03*	−0.38*	−0.73 to −0.03
Punishment Learning	Rich Choices (%)	58.70	9.92	62	64.32	10.86	63	0.003*	−0.54*	−0.89 to −0.18
Ultimatum Game, Proposer	Propose for Self ($)	5.47	1.33	64	5.66	1.46	64	0.45	−0.14	−0.48 to 0.21
Ultimatum Game, Responder	Accept for Self, minimum ($)	2.90	1.72	63	3.23	1.61	63	0.20	−0.20	−0.5 to 0.15
Future Expectations	Win Probability in %	52.00	27.75	56	63.51	21.23	62	0.01*	−0.47*	−0.83 to −0.10
Delay Discounting	Now Choices (%)	66.94	24.78	64	71.06	23.36	64	0.34	−0.17	−0.52 to 0.18
Willingness-to-Wait	High Persistence Block (in secs)	13.77	2.82	64	14.74	2.06	63	0.03*	−0.39*	−0.74 to −0.04
	Low Persistence Block (in secs)	12.83	3.54	63	12.87	3.98	64	0.96	−0.01	−0.35 to 0.34

*Note*. *p < 0.05.

For the remaining six decision-making measures, there were only small differences between the two groups (*d*’s < 0.27), with confidence intervals that include zero, and none of which reached statistical significance (*p*’s > 0.13). This includes willingness to wait in the low persistence condition (where the optimal strategy is to quit waiting if the reward does not arrive by a certain point), decisions in the ultimatum game, and choices in the risk tolerance, ambiguity tolerance, and delay discounting tasks.

A one way ANOVA showed that type of treatment (no treatment, medication, therapy, combination of medication and therapy) did not impact performance on any of the decision tasks within the depressed group (*p*’s > 0.25).

### Factor analysis

To assess whether the differences we observed between MDD and controls reflected a single or multiple dimensions of decision-making, we used both factor analytic and multiple regression approaches. An exploratory factor analysis of the decision-making battery suggested multiple dimensions of decision making, some of which were associated with depression. Factor analysis identified four factors explaining 39.10% of the variance (Table 3). The first factor, which we have labeled Learning, consisted of reward and punishment reversal learning, delay discounting and responses in the Ultimatum Game. The second factor, which we have labeled Persistence, included the two conditions of the persistence task. The third factor, which we have labeled Uncertainty, consisted of ambiguity and risk tolerance. The fourth factor, which we have labeled Expectations, included future expectations and proposals in the Ultimatum Game. Though this factor solution picks up on some variance due to task features, as the learning tasks load on the same dimension as do the two measures of the persistence task, these are not the sole determinants of the factor solution, since the three preferential choice tasks load onto different dimensions as do proposals and responses in the Ultimatum Game.

**Table 3:** Factor loadings and communalities based on a principal axis factoring analysis with promax rotation for ten value-based decision-making tasks (N = 115).

	Learning Factor	Persistence Factor	Uncertainty Factor	Expectations Factor	Communality
Reward: Rich Choice	0.67				0.52
Punishment: Rich Choice	0.66				0.47
Now Choices	−0.54				0.30
Accept for Self	−0.38				0.14
High Persistence Cond.		0.79			0.68
Low Persistence Cond.		0.71			0.44
50–50 Choices			0.88		0.77
Safe Choices			0.43		0.19
Winning Probability			0.51	0.24	
Propose for Self			0.40	0.16	

*Note*. Factor loadings < 0.2 are suppressed.

We used logistic regression to determine which of these factors were associated with depression. The four dimensions together reliably distinguished depressed individuals from controls (Table 4; χ^2^ = 14.35, df = 4, *p* = 0.006, R^2^ = 0.16). The Learning factor, which weights reward and punishment learning most heavily, was a significant predictor (*p* = 0.03). The Expectations factor, which weights future expectations most heavily, was also a significant predictor (*p* = 0.01). The Persistence factor, which weights willingness-to-wait in the high persistence condition most heavily, was only marginally significant (*p* = 0.085).

**Table 4:** Summary of Logistic Regression Analysis for Decision Factors Predicting MDD and Healthy Controls.

	Full Logistic Regression Model	
	B(SE)	Sig.
Learning Factor	0.53(0.24)	0.03*
Persistence Factor	0.41(0.24)	0.09^
Uncertainty Factor	−0.001(0.25)	0.99
Expectations Factor	0.87(0.35)	0.01*
Constant	0.09(0.20)	0.64

Note. *p < 0.05, ^^^p = 0.09.

### Logistic regression

Logistic regression analyses also suggested that different decision-making tasks explain unique variance in depression. A logistic regression analysis using all of the decision tasks reliably distinguished depressed individuals from controls (χ^2^ = 21.63, df = 10, *p* < 0.02, R^2^ = 0.23). Using a step-wise procedure, we created a reduced model (Table 5). The reduced model contained three predictors – punishment learning, willingness-to-wait in the high persistence block, and expected winning probability – that reliably distinguished depressed individuals from controls (χ^2^ = 15, df = 3, *p* = 0.002, R^2^ = 0.16). Considered individually, performance on punishment learning (*p* = 0.02) and expected winning probability (*p* = 0.05) made a significant contribution to prediction, while the effects of willingness-to-wait in the high persistence block (p = 0.13) were not significant (Table 5).

**Table 5:** Summary of Logistic Regression Analysis for Decision Variables Predicting MDD and Healthy Controls.

	Full Logistic Regression Model		Reduced Logistic Regression Model	
	B(SE)	Sig.	B(SE)	Sig.
Safe Choices	0.01(0.01)	0.57		
50–50 Choices	0.00(0.01)	0.69		
Reward: Rich Choice	1.70(2.54)	0.50		
Punishment: Rich Choice	5.58(2.53)	0.03*	4.5(1.99)	0.02*
Propose for Self	−0.05(0.16)	0.78		
Accept for Self	0.22(0.13)	0.11	—	—
Winning Probability	0.02(0.01)	0.09^	0.02(0.01)	0.05*
Now Choices	0.01(0.01)	0.24		
High Persistence Cond.	0.21(0.12)	0.09^	0.14(0.09)	0.13
Low Persistence Cond.	−0.07(0.07)	0.30		
Constant	−8.79(2.70)	0.00	−5.69(1.85)	0.002

Note. *p < 0.05, ^^^p < 0.1.

### Out-of-sample prediction

Finally, we assessed the ability of decision-making tasks to predict depressed status out of sample. The leave-one-subject-out cross-validation prediction accuracy via boosted trees was 71.9%. Accuracy was 64.1% within the depressed group and 79.7% within the control group. The variables of highest importance were winning probability expectations (0.0159), followed by willingness to wait in the high persistence condition (0.0047) and punishment learning (0.0038). A variable with high variable importance measure basically means that the misclassification rate (as measured by Gini’s diversity index) goes up by a lot without that variable.

### Correlations

We assessed several self-report constructs relevant to depression. For exploratory purposes, we report the correlations between self-report measures and decision-making tasks, controlling for depressed status. Given the large number of comparisons, none of these correlations were statistically significant after correction for multiple comparisons (Table 6).

**Table 6:** Pearson Correlations Between Performance on Decision Tasks and Self-Report Questionnaires.

	Depression Scale	Anxiety Scale	Depression Anxiety Stress Scale (Dass			Anhedonia Scale	Self Esteem Scale	Behavioral Inhibition and Approach Scale (BIS/BAS)				Cognitive Behavioral Avoidance Scale (CBAS)			
	BDI	BAI	Stress Dass	Depression Dass	Anxiety Dass	Snaith Hamilton	RSES	BIS	Drive BAS	Fun Seeking BAS	Reward Responsiveness BAS	BS Total	BN Total	CNS Total	CS Total
Safe Choices	−0.03	−0.06	0.02	0.11	0.03	−0.03	−0.07	0.11	−0.01	0.03	0.04	−0.10	0.08	0.07	0.05
N	125	122	124	125	125	124	123	125	125	125	125	124	124	124	124
50–50 Choices	−0.11	−0.08	−0.08	0.01	−0.04	0.09	0.09	−0.04	0.18	0.03	0.12	−0.10	−0.10	−0.03	0.02
N	124	122	124	125	125	124	123	125	125	125	125	124	124	124	124
Reward: Rich Choice	0.10	0.07	0.08	0.09	−0.02	0.01	0.04	−0.01	0.02	0.03	−0.04	−0.04	0.01	−0.09	−0.03
N	125	122	124	125	125	124	123	125	125	125	125	124	124	124	124
Punishment: Rich Choice	−0.06	−0.09	−0.09	−0.10	0.05	−0.08	−0.04	−0.03	0.04	−0.13	−0.08	0.01	−0.07	−0.02	0.03
N	125	122	124	125	125	124	123	125	125	125	125	124	124	124	124
Propose for Self	0.05	0.09	0.01	−0.07	0.01	−0.07	0.04	−0.12	−0.07	0.04	0.00	−0.09	−0.03	−0.08	−0.18
N	125	122	124	125	125	124	123	125	125	125	125	124	124	124	124
Accept for Self	0.10	−0.11	0.02	0.02	−0.02	0.07	−0.02	−0.02	0.01	0.09	0.14	0.04	0.02	0.00	0.05
N	123	120	122	123	123	122	121	123	123	123	123	122	122	122	122
Winning Probability	−0.04	0.08	−0.03	0.09	0.06	0.05	0.07	−0.02	0.06	0.11	0.09	−0.04	−0.03	0.02	0.02
N	125	122	124	125	125	124	123	125	125	125	125	124	124	124	124
Now Choice	0.24*	0.18*	0.25*	0.28*	0.29**	−0.11	0.02	0.08	−0.06	−0.06	−0.05	0.26*	0.30**	0.34**	0.30**
N	125	122	124	125	125	124	123	125	125	125	125	124	124	124	124
High Persistence	−0.04	−0.01	0.01	0.17	0.01	0.06	−0.02	−0.04	0.03	0.11	−0.01	−0.12	−0.09	−0.07	−0.04
N	124	121	123	124	124	123	122	124	124	124	124	123	123	123	123
Low Persistence	−0.10	−0.03	−0.09	−0.04	−0.10	0.04	−0.03	−0.06	0.01	−0.05	0.07	−0.14	−0.11	−0.12	−0.09
N	124	121	123	124	124	123	122	124	124	124	124	123	123	123	123

*Note*. *p < 0.05, **p < 0.001. None of the correlations are significant after correcting for multiple comparisons. BDI = Beck Depression Inventory, BAI = Beck Anxiety Inventory, RSES = Rosenberg Self Esteem Scale, BIS = Behavioral Inhibition Scale, BAS = Behavioral Approach Scale, BS = Behavioral Social, BN = Behavioral Non Social, CNS = Cognitive Non Social, CS = Cognitive Social.

## Discussion

The first question this study addressed was on what tasks depressed individuals show the largest differences in value-based decision making. We observed medium effects sizes for differences between depressed individuals and healthy controls in punishment and reward learning, future expectations and persistence. The differences in punishment learning and future expectations were the largest, and survived statistical correction for multiple comparisons. Depressed individuals were less successful at learning from punishments and believed they had a lower probability of winning additional money (*i.e*., receiving a positive outcome from the experiment). There were only small differences, which were not statistically significant, between depressed individuals and healthy controls in their degree of delay discounting, risk tolerance, ambiguity tolerance, performance in the ultimatum game, and in limiting persistence when this was the best strategy. Though many of these tasks have been studied individually before, our approach allows the size of the effects across tasks to be more directly compared, and also helps address recent concerns about replicability in psychological science^49,50^, which highlight the need to better understand which effects (in this case, differences between depressed and non-depressed) are reliable and replicable and which are not.

The second question this study addressed was whether differences in value-based decision making should be conceptualized as effects on a single dimension of decision making or on multiple dimensions. The four tasks that showed the largest differences were reward and punishment learning, expectations about future positive events, and persistence in waiting for delayed rewards. The latter three tasks (*i.e*., excluding reward learning, where performance was correlated with punishment learning) loaded onto three separate dimensions in a factor analysis of decision performance. In a logistic regression, the two factors that weighted most heavily punishment and reward learning (Learning Factor) and future expectations (Expectations Factor) each accounted for distinct variance in predicting depression. When using the individual tasks rather than the four factors, punishment learning and future expectations each accounted for distinct variance in the final, reduced model. Thus both regression and factor analytic approaches suggest that decision-making deficits in depression are multidimensional.

The third question this study addressed was how accurately individuals could be categorized as depressed based on decision making alone. Using machine learning techniques, we could predict depressed status out-of-sample with 71.9% accuracy. Although not dramatic, this degree of accuracy was significantly better than chance and comparable to recent out-of-sample prediction accuracies achieved with machine learning from Facebook language^51^. Of course, we would achieve higher accuracies if we used the self-report measures designed to assess different aspects of the current diagnostic criteria for depression. However, our interest in examining the predictive accuracy of decision-making tasks is not to develop a screening test for depression according to current diagnostic criteria, but rather to identify theoretically-driven behavioral measures that capture some of the heterogeneous differences between depressed and healthy individuals. Considering this goal, the predictive accuracy of value-based decision making tasks we observed shows promise for future research in this area.

Interestingly, three of the tasks that were most sensitive to depression in our study – reward and punishment learning and probability judgments – have been associated with depression previously. There is now a large body of research showing that reward and punishment processing is impaired in mood disorders^5,52–55^. For example, adults with MDD develop weaker response biases in perceptual categorization or memory tasks under conditions where the probability of reward is higher following one response versus the other^56,57^. This decreased reward bias persists during remission^19^ and predicts worse outcomes during treatment. Depressed individuals are also often impaired at the Iowa Gambling Task, which requires learning to make advantageous choices from reward and punishment feedback^58–60^. Previous studies using instrumental reversal learning tasks more similar to the ones we used here, however, have yielded more equivocal findings^53,61–65^. None of these previous studies of reversal learning, though, have involved learning to avoid punishments, and our findings suggest that reinforcement learning in the context of punishment is the domain in which depressed individuals differ most from controls. Given the well-established neurobiology of reinforcement learning, our results suggest that changes in dopaminergic neural circuits that signal prediction errors or in their target regions in the ventromedial frontal lobe and ventral striatum that signal predicted reward value may play a critical role in depression^66,67^.

Depressed also differed reliably from healthy individuals in future expectations, estimating a significantly lower likelihood of winning additional payment, even though the true likelihoods did not differ across the two groups. As the depressed group predicted a 50% probability on average, when the true probability was much higher, they exhibited a clear pessimistic bias. This aligns with previous research showing that depressed individuals were more pessimistic in their predictions of the likelihood of future outcomes than non-depressed individuals, given identical information with which to make their forecasts^23,25,68,69^. That this difference was apparent using only one question in the current study suggests its robustness, and indeed there is a long history of research investigating pessimistic future expectations in depression^69,70^. Given that individual differences in optimism are associated with neural activity in the ventromedial frontal lobe, this further supports the idea that abnormalities in this region play an important role in depression^71^.

Depressed individuals were also less willing to continue waiting for delayed rewards under conditions where such persistence is optimal. As this task has not been studied in depression previously and these differences were no longer significant after correction for multiple comparisons, further replication is needed before drawing strong conclusions. That willingness to wait was only a marginal predictor of depression in both the factor and task regression analyses is another indicator that caution is warranted. However, given that frustration, time pressure and irritability are often features of depression and that the firing of serotonin neurons is correlated with continued waiting^72,73^, further investigation of persistence in depression is clearly merited. An interesting question is whether performance on persistence tasks may be related to the reductions in motivation observed in depressed individuals in effort discounting tasks^74–77^.

We did not find differences between depressed and non-depressed individuals in delay discounting, risk or ambiguity tolerance, or social bargaining in the ultimatum game. These results are largely consistent with previous findings. In several previous studies, depression alone did not have a main effect on temporal discounting^27,78,79^. Though some reports have found an increased^24,80,81^ or decreased^82^ likelihood of rejecting unfair offers in the ultimatum game in depressed individuals, studies that have assessed social bargaining in larger samples (total n > 50) have found, similar to ours, no differences in the rejection of unfair proposals^18,83–86^. Though ours is the first assessment of risk and ambiguity preferences in depression as these constructs are measured in experimental economics, our results are consistent with previous studies showing no differences in risky decision making in depressed individuals in tasks with trial-by-trial feedback^16,22,87^.

These results should be interpreted in light of a few limitations. Although we did screen out individuals with bipolar, substance abuse and/or psychotic symptoms, we did not account for other co-morbidities. Beyond the screen for substance use disorder, we did not collect detailed information on alcohol use or smoking, which are associated with performance on some decision-making tasks. Although the two groups were matched in IQ and demographics, there may be other, unmeasured, confounds, such as more specific cognitive measures like working memory. As the mean BDI scores in our depressed group was on the border between moderate and severe depression, our conclusions may be most applicable to individuals with depression of this severity. Finally, given our cross-sectional design, we could not examine effects of drug or therapy treatments on decision making.

These limitations withstanding, we see two important directions for extending this work. First, the current study focused on distinguishing depressed from healthy individuals. An equally important task is to distinguish different mental illnesses from each other and/or discover their commonalities. We know very little about whether decision-making measures may prove useful for this purpose, and work using decision-making tasks as transdiagnostic dimensions should clearly be a priority for the future.

Second, the decision measures that were most predictive in the current study may prove even more so with further refinement. Our study provides an important first step in identifying the behavioral tasks where there are the largest differences, and to do so we focused on basic metrics of performance that would be easily replicable. However, a critical next step in a computational psychiatry approach will be to use computational modelling to more precisely identify the source of performance differences in reward and punishment learning and optimal persistence^88^. Beyond refining the behavioral measures, reward and punishment learning, future expectations and persistence are all associated with distinct neural circuits, and therefore may point towards brain measures that could prove even more sensitive and specific. Overall, further investigation of decision making in clinically depressed individuals could lead to a better understanding of the substantial heterogeneity within individuals with this diagnosis and could then further enable stratification to treatment based on decision-making profiles.

## Data Availability

The data that support the findings of this study are available from the corresponding author upon reasonable request.
